# SEX DIFFERENCES IN SKELETAL MUSCLE ENERGETICS AMONG OLDER ADULTS

**DOI:** 10.1093/geroni/igac059.2914

**Published:** 2022-12-20

**Authors:** Philip Kramer, Giovanna Distefano, Sofhia Ramos, Bret Goodpaster, Paul Coen, David Marcinek, Peggy Cawthon, Anthony Molina

**Affiliations:** Wake Forest University School of Medicine, Winston-Salem, North Carolina, United States; Translational Research Institute for Metabolism and Diabetes, AdventHealth, Orlando, Florida, United States; AdventHealth, Orlando, Florida, United States; Translational Research Institute for Metabolism and Diabetes, AdventHealth, Orlando, Florida, United States; AdventHealth, Orlando, Florida, United States; University of Washington, Seattle, Washington, United States; University of California, San Francisco, San Francisco, California, United States; UC San Diego School of Medicine - Division of Geriatrics, Gerontology, and Palliative Care, La Jolla, California, United States

## Abstract

Mobility impairment is the most common disability among older adults, with an earlier presentation, higher prevalence, and greater severity in women compared to men. A decline in skeletal muscle metabolism contributes to the loss of mobility with age. However, it is unknown if sex-specific differences in muscle energetics can explain the disparity in mobility impairment among men and women. In the Study of Muscle, Mobility, and Aging (SOMMA), muscle energetics was characterized using in vivo Phosphorus Magnetic Resonance Spectroscopy and High-Resolution Respirometry of permeabilized fiber bundles from muscle biopsies. In this analysis of 773 participants aged 70–94 years, 519 were women, of which 16% were deemed lower extremity mobility impaired based on a Short Physical Performance Battery (SPPB) score

